# Case of IgG4-Related Disease Presenting With Dissecting Aneurysms in the Bilateral Internal Iliac Arteries After the Resolution of Periarteritis

**DOI:** 10.7759/cureus.77052

**Published:** 2025-01-07

**Authors:** Hiroaki Hagiwara, Norihisa Karube, Megumi Amano, Mizuki Ikawa, Yuji Uzawa

**Affiliations:** 1 Radiology, Yokohama Minamikyosai Hospital, Yokohama, JPN; 2 Cardiovascular Surgery, Yokohama Minamkyosai Hospital, Yokohama, JPN; 3 Rheumatology, Yokohama Minamikyosai Hospital, Yokohama, JPN

**Keywords:** dissecting aneurysm, endovascular stent graft, igg4-related disease (igg4-rd), internal iliac artery aneurysm, periarteritis

## Abstract

In this report, we present the case of an 83-year-old man diagnosed with IgG4-related disease (IgG4-RD) who developed dissecting aneurysms in the internal iliac arteries post-resolution of bilateral periarteritis. The patient initially presented with weight loss, anemia, and lymphadenopathy for over 13 years without a definitive diagnosis. Laboratory tests revealed elevated serum IgG4 levels and decreased complement levels, leading to a diagnosis of IgG4-RD. This was confirmed by a biopsy of the left axillary lymph node, which showed dense infiltration of IgG4-positive plasma cells.

Contrast-enhanced CT revealed extensive periarterial soft tissue masses around the thoracic aorta, thoracic vertebrae, mediastinum, and internal iliac arteries, which were significantly reduced after the initiation of corticosteroid therapy with high dose prednisolone. Despite the initial improvement in clinical and radiological findings, the patient subsequently developed dissecting aneurysms in both internal iliac arteries. The right internal iliac artery aneurysm exhibited rapid enlargement, necessitating endovascular treatment with stent grafts to prevent rupture.

This case highlights the complexity of vascular involvement in IgG4-RD, underscoring the need for careful monitoring even after apparent disease improvement. Although arterial wall thickening improves, the infiltration of IgG4-positive plasma cells persists indicating that the risks of aneurysm and arterial dissection remain. This is the sparsely reported case of bilateral dissecting aneurysms of the internal iliac arteries associated with IgG4-RD, providing new insights into the vascular manifestations and management of IgG4-RD.

## Introduction

IgG4-related disease (IgG4-RD) is a systemic inflammatory condition characterized by the infiltration of IgG4-positive plasma cells into various tissues, causing fibrosis and organ dysfunction. Although it commonly affects organs, such as the pancreas, salivary glands, and lymph nodes, vascular involvement is an important and less commonly recognized aspect of the disease.

Arterial involvement in IgG4-RD primarily manifests as aortitis, periarteritis, aneurysm, and stenosis [1]. Inflammation of the arterial walls leads to thickening, causing aneurysm formation due to weakening of the vessel walls or stenosis due to fibrotic narrowing.

Aneurysms in IgG4-RD are characterized by dense infiltration of IgG4-positive plasma cells and fibrosis, predominantly affecting large vessels such as the abdominal aorta and iliac arteries. These aneurysms have a heightened rupture risk due to the compromised structural integrity of the vessel wall [2]. Herein, we report a case in which dissecting aneurysms developed following the regression of periarteritis of the bilateral internal iliac arteries.

## Case presentation

**Figure 1:**
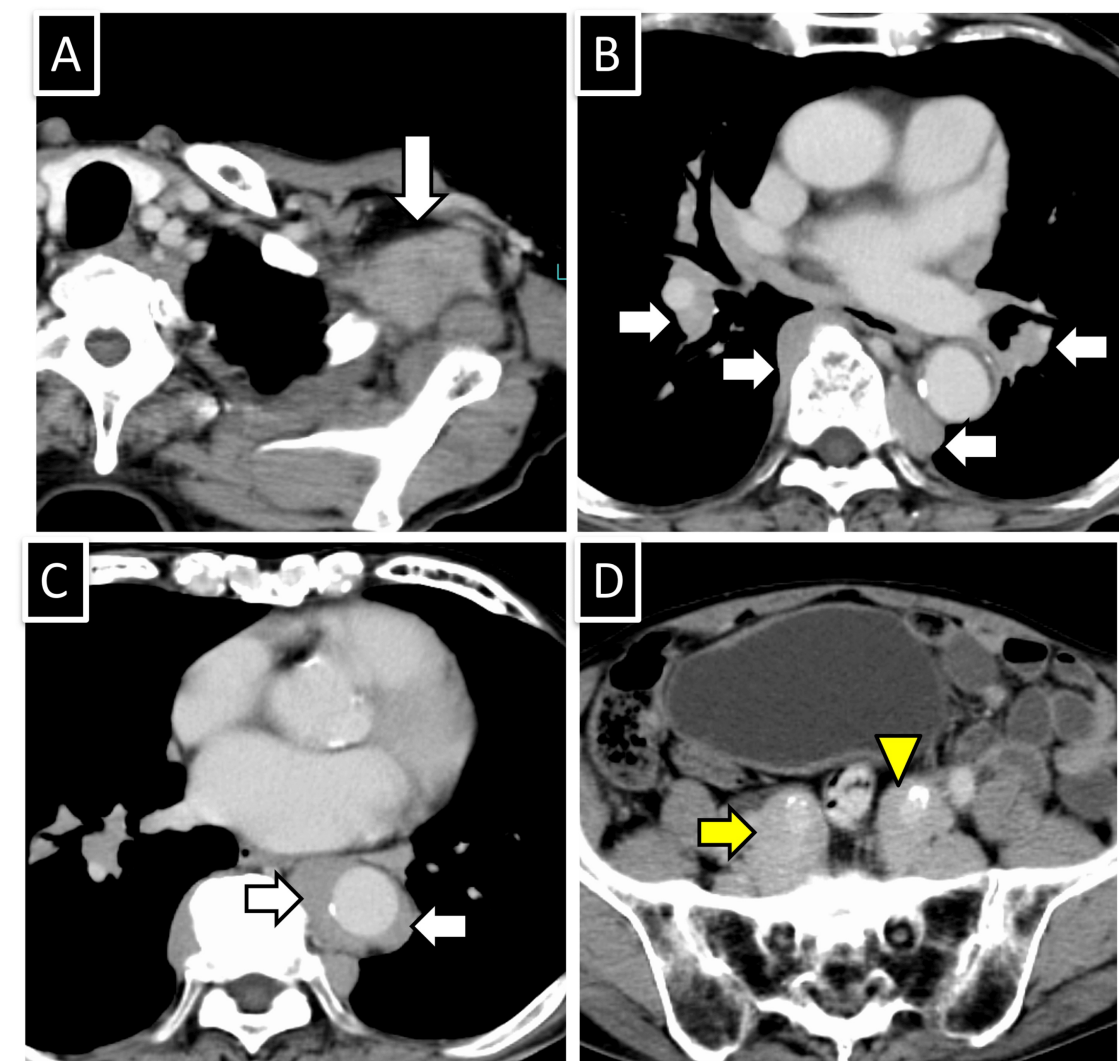
Contrast-enhanced CT before treatment. (A) Enlarged lymph node in the left axilla (arrow). (B) Soft tissue density masses observed in the hilum and paravertebral thoracic region (arrow). (C) Soft tissue density mass surrounding the descending aorta (arrows). (D) Soft tissue density masses around both internal iliac arteries (yellow arrow and yellow arrowhead). Atherosclerosis-induced calcification was observed in internal iliac arteries.

An 83-year-old man visited his previous physician 13 years ago with chief complaints of weight loss, anemia, and lymphadenopathy. He was diagnosed with suspected Castleman disease. Lymphadenopathy was spontaneously reduced, and the patient was under observation without treatment by his previous physician. His medical history included hypertension and atrial fibrillation. The patient experienced dyspnea due to worsening anemia and was referred to our hospital for further evaluation. Blood tests revealed elevated IgG4 levels (1,910 mg/dL, normal range 4.8-105.0 mg/dL), decreased complement C3 levels at 26.8 mg/dL (normal range: 73-138 mg/dL), C4 at 2.3 mg/dL (normal range: 11-31 mg/dL), and increased urinary B2-MG at 0.80 mg/L (normal range: ≤0.29 mg/L) (Table 1). Whole-body contrast-enhanced CT showed lymphadenopathy in the left axilla (Figures 1A-1D). Soft tissue masses were observed around the descending thoracic aorta, thoracic vertebral bodies, mediastinum to the hilum, and both internal iliac arteries.

**Table 1:** Laboratory investigations

Tests	Results before treatment	Results 1-year post-treatment initiation.	Results 1.5-year post-treatment initiation.	Normal ranges
IgG4	1,910 mg/dL	142 mg/dL	200 mg/dL	4.8-105.0 mg/dL
C3	26.8 mg/dL	121 mg/dL	120 mg/dL	73-138 mg/dL
C4	2.3 mg/dL	39 mg/dL	38 mg/dL	11-31 mg/dL
Urinary B2-MG	0.80 mg/L	0.07 mg/L	0.17 mg/L	≤0.29 mg/L

A biopsy of the left axillary lymph node revealed dense infiltration of IgG4-positive plasma cells, leading to the diagnosis of IgG4-RD. The IgG4/IgG ratio was approximately 80%, and characteristic storiform fibrosis was observed. However, there was no evidence of obliterative phlebitis. Treatment was initiated with prednisolone (35 mg, OD). Following treatment initiation, IgG4 levels decreased to 99.1 mg/dL.

**Figure 2:**
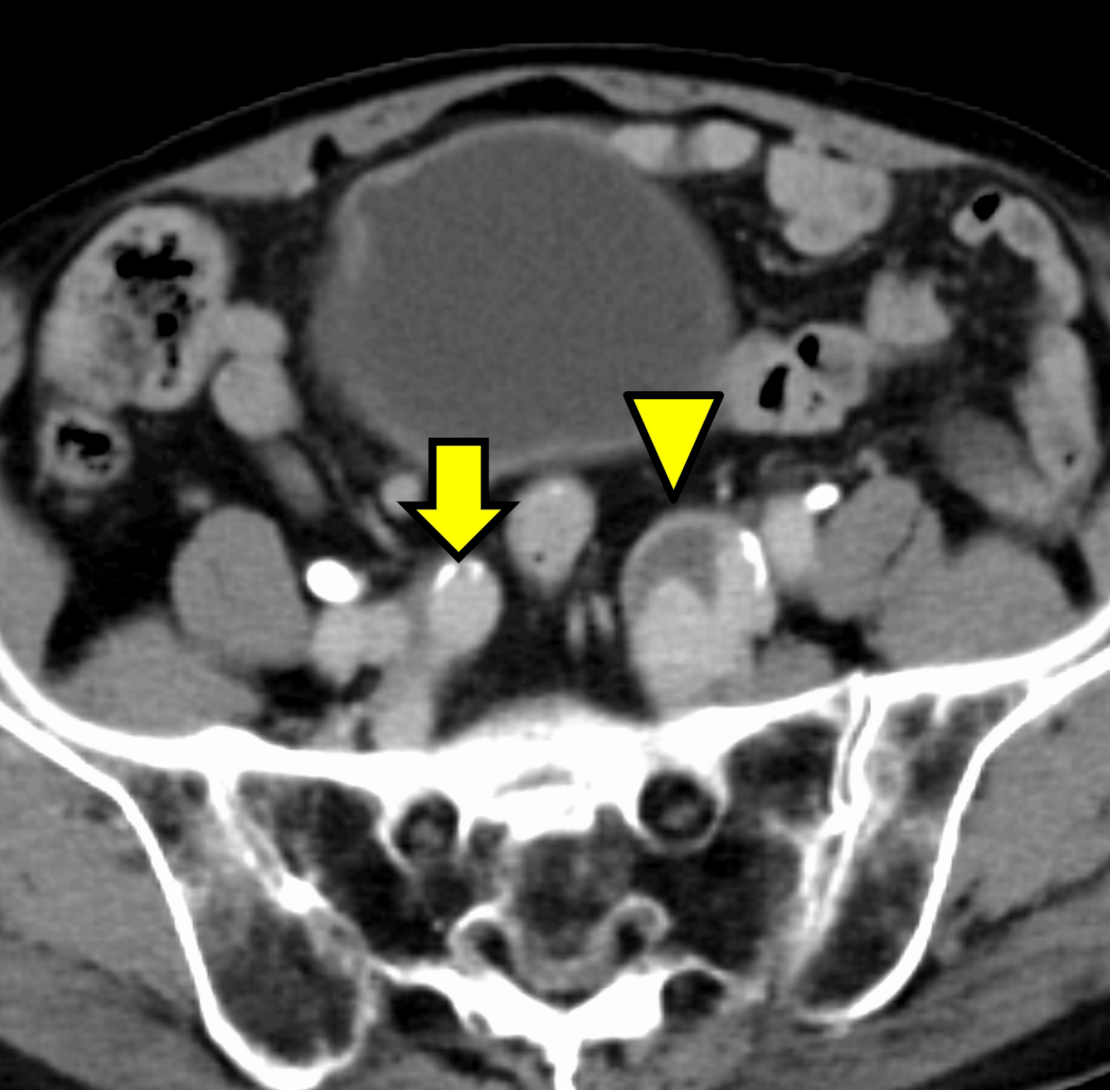
Contrast-enhanced CT 1-year post-treatment initiation. The soft tissue mass around the right internal iliac artery disappeared (yellow arrow), and a dissecting aneurysm appeared in the left internal iliac artery (yellow arrowhead).

One year after the initiation of treatment, the dose of prednisolone was gradually reduced to 10 mg per day, and the test results had also shown improvement. Contrast-enhanced CT revealed a significant reduction in all previously observed lesions, with the lesion around the right internal iliac artery disappearing completely (Figure 2). However, a dissecting aneurysm appeared in the left internal iliac artery with partial thrombosis of the false lumen. The patient had no episodes of abdominal pain or other symptoms due to arterial dissection.　There were no symptoms or test results to suggest infection.

**Figure 3:**
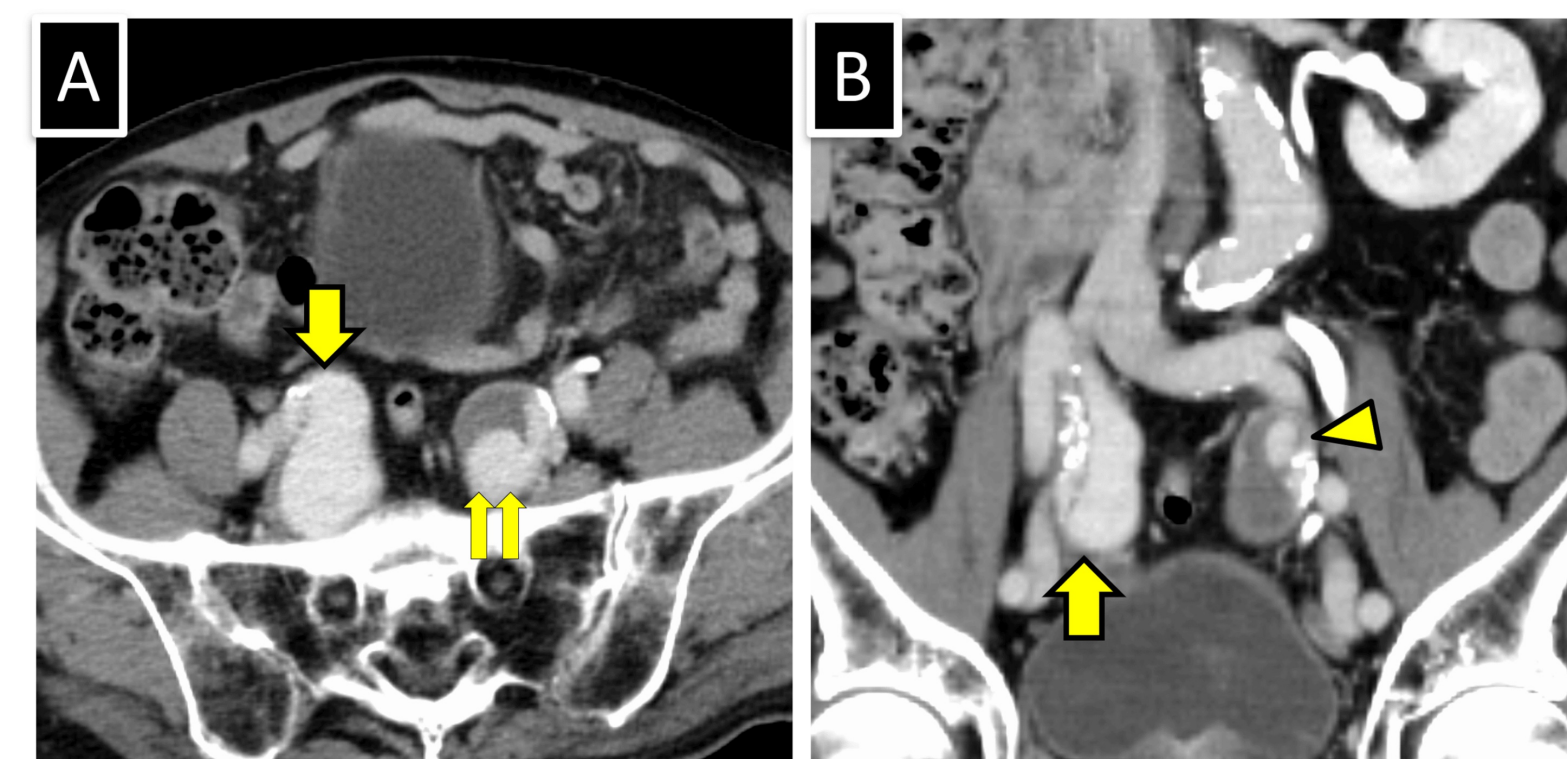
Contrast-enhanced CT 1.5 years post-treatment initiation. (A) In an axial image, a new dissecting aneurysm appeared in the right internal iliac artery (yellow arrow). No significant change was observed in the dissecting aneurysm in the left internal iliac artery, which had partially thrombosed, compared to six months ago. Two patent false lumens are visible: one large (yellow double arrow) and one small lumens. (B) In the coronal image, the right false lumen is not thrombosed (yellow arrow) and true lumen is narrowed due to displacement by intimal calcification. Similarly, the true lumen on the left is narrowed, with a small patent false lumen depicted on the cranial side (yellow arrowhead).

Six months later, prednisolone was tapered to 7 mg per day. CT showed no significant changes in the left internal iliac aneurysm; however, a dissecting aneurysm appeared in the right internal iliac artery (Figures 3A, 3B). This aneurysm was larger than the left-sided lesion and lacked thrombosis in the false lumen, indicating rapid enlargement.

**Figure 4:**
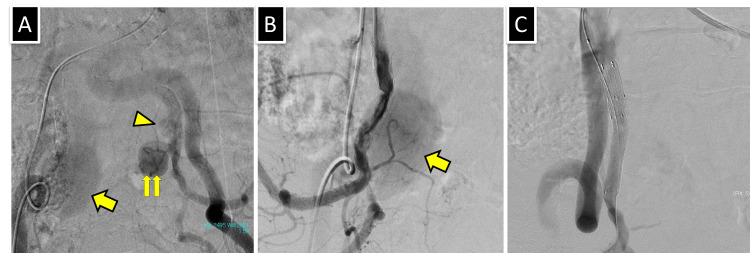
Endovascular therapy. (A) Aortography shows a large patent false lumen of the dissecting aneurysm in the right internal iliac artery (yellow arrow). In contrast, the dissecting aneurysm in the left internal iliac artery reveals a small cranial (yellow arrowhead) and large patent false lumens (double arrow). (B) An iliac arteriography demonstrated the false lumen(yellow arrow) and narrowing of the proximal true lumen. (C) After placing a covered stent in the right internal iliac artery, the depiction of the false lumen disappeared and narrowing of the true lumen improved.

Endovascular therapy was planned to prevent aneurysm rupture and was performed through the left common femoral artery by crossover approach with a 7Fr guiding sheath (Parent Cross; Medikit, Tokyo, Japan) under local anesthesia. Aortography revealed a large false lumen in the right internal iliac artery aneurysm (Figure 4A). The false lumen of the left internal iliac aneurysm was divided into small cranial and large caudal parts. Arteriography of the right internal iliac artery revealed a narrowing of the true lumen (Figure 4B). A 0.018-inch stiff wire (V-18, Boston Scientific, Natick, MA) was used for stent graft delivery. An 8mm in diameter and 50mm in length stent graft (Viabahn, Gore, Flagstaff, AZ) was advanced until it covered the entry site of the dissection and was then deployed. Then, due to a residual endoleak, an additional 8mm in diameter and 25mm in length Viabahn stent graft was placed proximally, closing the false lumen (Figure 4C).

**Figure 5:**
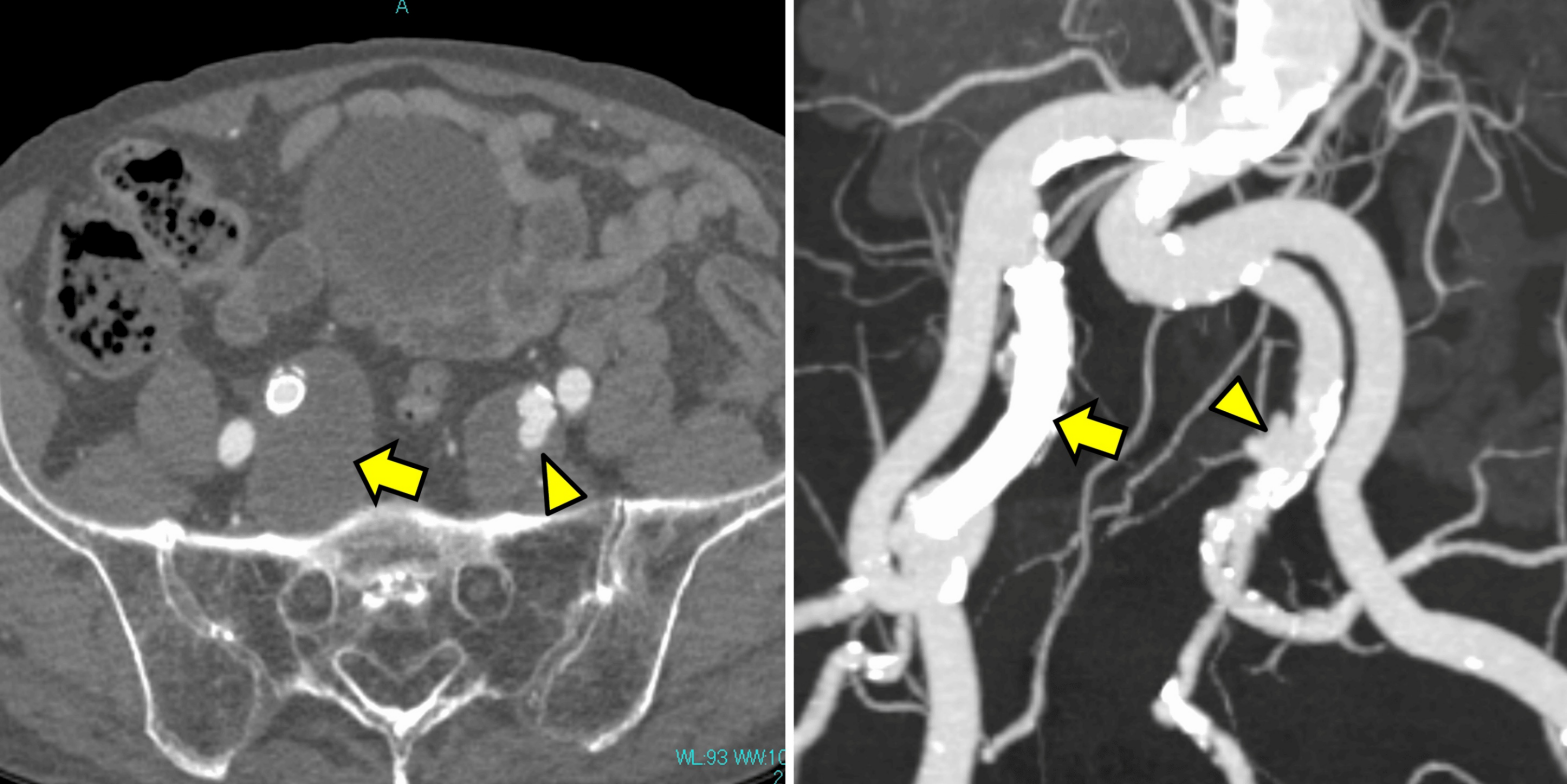
Contrast-enhanced CT five months after endovascular therapy. (Left) The small cranial false lumen of the left internal iliac aneurysm remained, similar to the previous scan (yellow arrowhead), whereas the large caudal false lumen was spontaneously thrombosed and occluded. (Right) The stent placed in the right internal iliac artery remains patent, and the false lumen of the aneurysm is completely thrombosed and occluded (yellow arrow).

Six months postoperatively, contrast-enhanced CT showed that the false lumen in the right internal iliac artery was thrombosed and occluded, and the stent remained well-expanded and patent (Figure 5). Likewise, the large caudal false lumen of the left internal iliac aneurysm spontaneously thrombosed and occluded; however, the small cranial false lumen persisted.

## Discussion

Periarteritis associated with IgG4-RD occurs predominantly in the abdominal aorta and its distal branches. According to Ozawa et al., 65 of 179 patients (36.3%) with IgG4-RD exhibited aortitis/periarteritis, which most commonly affects the abdominal aorta while extending to the iliac arteries [1]. Clinical parameters comparing IgG4-RD patients with and without aortitis/periarteritis revealed significantly higher disease activity in the former group, which was characterized by a higher age at onset and elevated levels of serum IgG, IgG4, circulating immune complexes, and soluble interleukin-2 receptors. Furthermore, corticosteroid therapy improved arterial wall thickening but exacerbated luminal dilation, with pretreatment arterial diameter dilatation identified as a risk factor [3]. The distribution of aneurysms in IgG4-RD varies, with coronary, thoracic, abdominal aortic, and iliac artery aneurysms being commonly reported [4-6]. These aneurysms are associated with active disease, as indicated by arterial wall thickening and luminal dilatation due to IgG4-positive plasma cell infiltration, which weakens the arterial wall. Additionally, steroid treatment may contribute to the progression of aneurysm [7]. However, post-treatment cases of splenic and iliac artery rupture were reported [2,8]. In the present case, corticosteroid treatment resolved the periarterial lesions and decreased IgG4 levels, indicating an improvement in the disease state. However, the occurrence of aneurysms suggests that disease improvement may not necessarily reduce the risk of aneurysm formation.

Aneurysms associated with IgG4-RD are primarily fusiform or saccular, with only a few dissecting aneurysms. Few reports on dissections of the coronary, carotid, and aortic arteries exist [9,10]. Cases of dissecting aneurysms of the internal iliac artery have been sparsely reported. Sugaya et al. reported a case of chronic aortic dissection in a patient whose condition improved with treatment [11]. Although the thickening of the ascending aorta was slight, pathological examination after aortic replacement surgery revealed infiltration of IgG4-positive plasma cells into the aortic adventitia, which was the primary reason underlying dissection. Herein, a similar mechanism led to the dissection of both internal iliac arteries.

Although dissecting aneurysms of the iliac arteries has been reported [12], bilateral cases remain undocumented. Bilateral internal iliac aneurysms, which are often attributed to atherosclerosis, are reported frequently [13,14]. Considering the presence of arterial calcification in other regions, a combination of IgG4-RD and atherosclerosis may have been implicated in our case.

Aneurysm treatment in patients with IgG4-RD involves surgical vascular replacement or stent placement, with emergency vascular replacement being necessary in cases of rupture [2,15]. However, treatment methods for non-ruptured cases remain controversial. Herein, the rapid enlargement of the right internal iliac aneurysm warranted stent placement for rupture prevention. Conversely, the large false lumen in the left internal iliac artery thrombosed spontaneously, suggesting that the right aneurysm may have been similarly occluded without intervention.

## Conclusions

Aneurysms in IgG4-RD are predominantly affecting large vessels such as abdominal aorta and iliac arteries. Most of these aneurysms are fusiform or saccular in shape, accompanied by thickening of the arterial wall. In this study, we documented the first case of a dissecting aneurysm that developed post-regression of bilateral periarterial lesions in a patient with IgG4-RD. Despite disease improvement and periarterial lesion regression, residual arterial fragility can result in aneurysm formation, suggesting the importance of vigilant monitoring of arterial lesions in patients with IgG4-RD.
